# High-Risk Siblings without Autism: Insights from a Clinical and Eye-Tracking Study

**DOI:** 10.3390/jpm12111789

**Published:** 2022-10-29

**Authors:** Valeria Costanzo, Antonio Narzisi, Sonia Cerullo, Giulia Crifaci, Maria Boncoddo, Marco Turi, Fabio Apicella, Raffaella Tancredi, Filippo Muratori, Sara Calderoni, Lucia Billeci

**Affiliations:** 1 Department of Developmental Neuroscience, IRCCS Fondazione Stella Maris, 56128 Calambrone, Italy; 2 Child Psychopathology Unit, Scientific Institute IRCCS Eugenio Medea, Via Don Luigi Monza 20, Bosisio Parini, 22040 Lecco, Italy; 3 Institute of Clinical Physiology, National Research Council of Italy (CNR), 56124 Pisa, Italy; 4 Interdepartmental Program “Autism 0-90”, “G. Martino” University Hospital of Messina, 98100 Messina, Italy; 5 Stella Maris Mediterraneo Foundation, 85032 Chiaromonte, Italy; 6 Department of Clinical and Experimental Medicine, University of Pisa, 56126 Pisa, Italy

**Keywords:** Autism Spectrum Disorder (ASD), eye tracking, initiating joint attention, high risk-noASD, atypical development

## Abstract

Joint attention (JA)—the human ability to coordinate our attention with that of other people—is impaired in the early stage of Autism Spectrum Disorder (ASD). However, little is known about the JA skills in the younger siblings of children with ASD who do not develop ASD at 36 months of age [high-risk (HR)-noASD]. In order to advance our understanding of this topic, a prospective multicenter observational study was conducted with three groups of toddlers (age range: 18–33 months): 17 with ASD, 19 with HR-noASD and 16 with typical development (TD). All subjects underwent a comprehensive clinical assessment and an eye-tracking experiment with pre-recorded stimuli in which the visual patterns during two tasks eliciting initiating joint attention (IJA) were measured. Specifically, fixations, transitions and alternating gaze were analyzed. Clinical evaluation revealed that HR-noASD subjects had lower non-verbal cognitive skills than TD children, while similar levels of restricted and repetitive behaviors and better social communication skills were detected in comparison with ASD children. Eye-tracking paradigms indicated that HR-noASD toddlers had visual patterns resembling TD in terms of target-object-to-face gaze alternations, while their looking behaviors were similar to ASD toddlers regarding not-target-object-to-face gaze alternations. This study indicated that high-risk, unaffected siblings displayed a shared profile of IJA-eye-tracking measures with both ASD patients and TD controls, providing new insights into the characterization of social attention in this group of toddlers.

## 1. Introduction

### 1.1. High-Risk Population for ASD

Recent epidemiological studies indicated that ASD prevalence is about 2.3% in the U.S. [1] and 1.15% in Italy [2]. This rate significantly increased among younger siblings of individuals with ASD, in which the overall risk of recurrence of ASD is estimated to be between 6.1% and 18.7% [3,4], with a higher recurrence risk in siblings of girls with ASD than in siblings of boys with ASD [5,6].

In this context, infant sibling studies have proliferated over recent years with the aim of providing not only the opportunity to learn about the earliest signs and symptoms of the ASD phenotype but also the chance to achieve an early diagnosis and intervention for affected children [7].

In addition to the higher prevalence of a diagnosis of ASD or of other neurodevelopmental disorders compared with the general population, siblings of children with ASD display a range of non-typical developmental outcomes that span several developmental domains, including language, cognition, social communication, motor skills and adaptive functioning [8,9,10]. Autistic traits below the clinical threshold are referred to as the ‘broader autism phenotype’ (BAP) (reviewed by [11]). For example, in a large, multicenter and longitudinal study [12], 28% of high-risk (HR) toddlers demonstrated, at 36 months of age, an atypical development (not including ASD) in cognitive, motor, receptive or expressive language and social communication. Another study [13] indicated that at 12 months of age, 19% of HR-noASD siblings showed increased social-communication difficulties, lower levels of cognitive functioning and more internalizing problems. In a large, multisite investigation involving 507 HR and 324 low-risk (LR) subjects, Messinger et al. [14] reported that, among HR siblings without an ASD diagnosis, the outcomes at 36 months: (i) 65% appeared typical as far as ASD symptoms and developmental functioning, (ii) 14% had high ASD severity and high levels of developmental functioning, (iii) 21% were characterized by either elevated levels of ASD severity (but still subthreshold) and low-average developmental functioning or low levels of ASD severity in the presence of lower developmental functioning. In addition, Miller et al. [15] demonstrated elevated rates of pragmatic language difficulties in HR-noASD at 36 months; in most cases, they did not have more general language impairments.

A research consortium study on infant siblings [16] reported mild-to-moderate levels of developmental delay in HR siblings who did not receive a diagnosis of ASD at 36 months. Moreover, the HR-noASD subjects had higher parent-reported levels of ASD symptoms and poorer adaptive functioning. Salomone and colleagues [10] followed the cognitive and adaptive skills of an HR population (HR-ASD and HR-noASD) from 7 months to 7 years of age and found lower cognitive skills over time in both HR-ASD and HR-noASD subjects compared with low-risk children. Of note, an increasing gap with age between the HR and LR subjects was detected, with the HR-ASD group showing lower skills compared with the LR and HR-noASD children at 7 years of age. Regarding the HR-noASD group, they had lower overall adaptive behavior skills than LR children, though significantly better than HR-ASD children.

Taken together, these findings highlighted the variability in the outcome but also the increased risk for a non-typical development among the younger siblings of children with ASD who do not meet the criteria for ASD. Research evidence suggests that this heterogeneity in outcomes may derive from a different constellation of early markers, emerging during the first two years of life and pushing the developmental path outside its typical course. Focusing on HR infants with a non-ASD outcome, some authors found differences also at an early age. For example, Wagner et al. [17] described that eye-tracking measurements reduced attention to faces—particularly the eyes and mouth—in HR-noASD infants compared with both LR- and HR-ASD infants at 6 months of age. In addition, this study revealed that early attention to the face and eyes was positively related to social behaviors at 18 months in the LR but not in the HR-noASD group, in which early face scanning is negatively related to the expressive language ability at 18 months, Webb et al. [18] also found that HR-noASD toddlers at 18–30 months spend more time to habituate to a face than TD toddlers without a sibling with ASD, similarly to ASD toddlers.

Hence, HR subjects seem to exhibit a different developmental trajectory and different endophenotypes even at an early age, regardless of whether they receive a formal diagnosis of ASD. Rankin and Tomeny [19] found that while ASD children and siblings with BAP share symptom commonalities, the structure of those symptoms may significantly differ in terms of a greater number of milder symptoms in siblings with BAP rather than fewer and more severe symptoms in ASD.

In this context, bio-behavioral markers may help to objectively describe not only different subtypes of ASD but also the subclinical profiles of the BAP. Eye tracking technology is considered a promising approach for identifying such bio-behavioral markers due to its non-invasiveness, ease of administration and interpretation, as well as its adjustability to the age and clinical features of the experimental population [20,21].

### 1.2. Eye-Tracking Studies Exploring Joint Attention

Several methodological approaches have recently been used to study ASD and its early features in the HR-ASD population, including the use of novel technologies, such as the eye-tracking [22], which enabled the study of visual direction performance by detecting the subject’s gaze with high accuracy. Acquisition in infancy is most commonly based on the reflection of near-infrared light from the cornea and the pupil [23]. The gaze position is calculated by computer algorithms based on video recordings collected by cameras placed in front of the observer. Since eye-tracking acquisition does not require advanced motor responses or language skills, it can be used for all ages and offers useful insights when studying ASD, HR–ASD infant and toddler populations [24].

Eye-tracking technology has been used to measure responding joint attention (RJA) and initiating joint attention (IJA), which are well documented as impaired in ASD (for a review, see [25]). While several studies focused on RJA, obtaining contrasting results [26,27,28,29], only a few analyzed IJA in ASD. In particular, a previous investigation [28] used pre-recorded stimuli in which a model seated at a table where a toy car suddenly started to move and detected more gaze alternations between the model and the car made by the ASD group than the TD group during IJA tasks.

More recently, Thorup et al. [30] used an eye-tracking paradigm to study IJA during live interactions between an examiner and 10-month-old LR and HR infants. The authors found a significant group difference in terms of the mean number of gaze alternations between the face and the object (in this case, lights), with lower values for the HR population. Moreover, reduced alternating gaze at 10 months was associated with more impairment in showing and pointing at 18 months of age in both the HR and LR groups, supporting the idea of using gaze alternations as an early measure of IJA.

The aim of the current study was to evaluate the visual patterns during IJA tasks in HR-noASD subjects compared to both ASD and TD toddlers by using video-based eye-tracking technology.

In our previous investigation comparing children with ASD and TD using the same eye-tracking paradigm as in this study [28], we observed that (i) children with ASD had more transitions from the target object to the face and (ii) TD children had more shifts from non-target object to face.

Although these findings may appear counterintuitive, we hypothesized that the higher transitions from target objects to face observed in children with ASD could be ascribed to the insistence on the sameness of these children so that they come back to the ‘known and predictable’ face more frequently than TD children. On the other hand, the lower transitions to non-target objects in ASD subjects could be due to deficits in divided attention, which might impair the ability to track more than one object on the scene in ASD individuals or to their difficulties in anticipation. Therefore, we proposed that TD children have higher attention to the non-target object because they foresee its possible movement.

Since previous studies have found that HR siblings who did not go on to have ASD had intermediate characteristics between the HR-ASD and LR groups [31,32], we hypothesized that the pattern of transitions of the HR-noASD toddlers would be in the middle between that of children with ASD and that of children with TD.

In the present study, we also explored alternating gaze measures because we hypothesized that they could be more powerful in detecting differences between groups than the measure of transitions [30].

## 2. Methods

### 2.1. Study Population

In this prospective study, a total of 52 children was enrolled from January 2012 to June 2018: 17 children diagnosed with ASD (mean age: 24.6 months, SD: 4.2; 13 boys and 4 girls), 19 high risk–non-ASD (HR-noASD) who were the younger siblings of children with ASD (mean age: 22.6 months, SD: 4.9; 12 boys, 7 girls) and 16 children with typical development (TD) having no first-degree relatives with ASD (mean age: 24.9, SD: 4.6; 10 boys, 6 girls). The sample age range was 18–33 months.

The ASD and the HR-noASD groups were enrolled in three different institutions: The Autism Unit of IRCCS Stella Maris of Pisa, the Division of Child Neurology and Psychiatry of the Academic Hospital of Messina and the Hospital of Matera. The clinical diagnosis of ASD was established according to the Diagnostic and Statistical Manual of Mental Disorders-Fifth Edition (DSM-5) criteria and confirmed using algorithm cut-offs on the Autism Diagnostic Observation Schedule-Second Edition (ADOS-2 [33]) and Autism Diagnostic Observation Schedule-Generic (ADOS-G [34]).

The exclusion criteria for the ASD group were as follows: neurological syndromes or focal neurological signs, significant sensory (visual or auditory) impairment, anamnesis of birth asphyxia, pre-term birth (<37 weeks), head injury or epilepsy, use of any psychotropic medication, potential secondary causes of ASD determined by Fragile-X analysis or screening tests for inborn errors of metabolism.

The exclusion criteria for the HR–noASD group were as follows: receiving a diagnosis of ASD at 36 months or obtaining a score above the diagnostic cut-off at the ADOS-2 (Toddler Module or Module 1) at the time of the assessment.

TD children were recruited from daycare centers in the Pisa, Messina and Matera metropolitan areas.

The demographics and clinical characteristics of the participants are shown in Table 1. The three groups (HR-noASD, ASD and TD) did not significantly differ in terms of gender (χ^2^ = 0.965, *p* = 0.617) and mean age (F = 1.312, *p* = 0.279). 

All parents provided written informed consent, including permission to use the video recordings for research purposes. The experimental procedures and the informed consent were approved by the ethics committee of the IRCCS Fondazione Stella Maris (Pisa, Italy).

### 2.2. Clinical Assessment

Children belonging to the three groups underwent a non-verbal developmental evaluation through the administration of the “Performance” subscale of the Griffiths Mental Developmental Scales-Extended Revised (GMDS-ER) [35,36], a standardized developmental assessment for children aged from birth to 8 years of age.

In addition, the ASD and HR-noASD subjects (14 out of 19 of the latter) underwent an Autism Diagnostic Observation Schedule evaluation (ADOS-G or ADOS-2) [33,34,37]. ADOS is a play-based, observer-rated assessment of ASD symptoms. We used the algorithms described by Gotham et al. [38,39,40] and Esler et al. [41] to derive an ADOS calibrated severity score (CSS) from a raw total, as well as a social affect domain calibrated severity score (SA-CSS) and Restricted and Repetitive Behaviors Domain Calibrated Severity Score (RRB-CSS) from raw domain totals. The use of CSS has been shown to result in a more uniform distribution across age and language levels and to be less influenced by non-ASD characteristics than raw scores [42].

Furthermore, the Vineland Adaptive Behavior Scales-Second Edition (VABS-II) [43] was used to evaluate the adaptive skills of 12 children with HR-noASD and 14 children with ASD.

The parents of 13 HR-noASD and 17 ASD children also completed the Social Communication Questionnaire (SCQ) Current [44,45] and the MacArthur Communicative Development Inventory (CDI)-Infant Form [46]. The SCQ Current is a 40-item questionnaire designed to screen for autism symptoms, whereas the CDI is a widely used parent-report measure of communicative and language development.

Table 1 summarizes the clinical characteristics of the three groups. In particular, HR-noASD toddlers showed a similar performance quotient (PQ) evaluated with GMDS-ER and similar Restricted and Repetitive Behaviors (RRB)-(CSS) of the ADOS (t = 1.85, *p* = 0.73) to ASD toddlers.

At the same time, HR-noASD toddlers showed better social and communicative skills, as measured with the SCQ Current version (t = 4.6, *p* = 0.0002), the Total CSS (t = 11.1, *p* = 0.0001) and Social Affect (SA)—CSS (t = 12.2, *p* = 0.0001) of the ADOS than ASD toddlers.

HR-noASD toddlers also showed better overall adaptive functioning, as measured with the overall Adaptive Behavior Composite Score (ABC) of the VABS-II (t = 3.42, *p* = 0.02) and a broader repertoire of gestures, comprehension and production vocabulary as measured with the MacArthur Communicative development inventory (number of words understood: t = 3.62, *p* = 0.001; number of words produced: t = 2.99, *p* = 0.005; number of gestures: t = 4.01, *p* = 0.0002) than ASD toddlers.

### 2.3. Stimuli and Procedure

All subjects underwent eye-tracking experiments using the same procedure and stimuli, as previously reported by Billeci et al. [28].

The experiment consisted of three tasks: one for responding to JA and two tasks for IJA, of which one was with a predictable event (JA-1) and the other with an unpredictable event (JA-2), respectively. Each task involved three segments: looking down (2 s), interaction (2 s) and joint attention (4 s for responding to JA and 7 s for initiating JA-1 and JA-2).

All the tasks took place in a common setting with a black background and a woman on screen sitting behind a black table. In this study, we focused only on the IJA tasks because our previous eye-tracking study [28] did not detect impairment in the RJA task in toddlers with ASD and because impairment in the ability to initiate joint attention is considered a hallmark feature of ASD (e.g., [47,48,49,50]).

In the JA segment of the first initiating JA task (IJA-1), one of the two cars (target object) started moving on the screen toward the other car (non-target object) until it reached approximately the center of the screen, while the model kept a direct gaze towards the camera maintaining a neutral, impassive facial expression. In the JA segment of the second initiating JA task (IJA-2), a toy truck (target object) unexpectedly appeared from off-screen and crossed the desk toward the opposite side while the model once again maintained a direct gaze toward the camera and a neutral, impassive facial expression. This non-interactive facial expression was chosen to ensure that child’s eye gaze toward the model’s face was not in response to any prompting by the model.

The duration of the whole task was 6 min. All the trials were presented in a block design paradigm. Each block consisted of four repetitions of one task, and the sequence of the blocks was always IJA-1 and then IJA-2. Each trial was preceded by a colorful “attention-getter” that was displayed at the center of the screen until the child looked at it for at least 500 milliseconds. This served to refocus the eyes before beginning the trial. Once attention was secured, the pre-recorded video replaced the attention-getter. Some trials were excluded on the basis of the criteria adopted by Bedford et al. [26]. The trial exclusion criteria were as follows:

- No looking at the model’s face during the interactive segment, which could be considered a prerequisite for JA behavior;
- Looking away from the computer screen for the entire JA phase.

The toddlers’ gazes were recorded by means of the SMI Eye Tracking device provided by SensoMotoric Instruments (SMI; Teltow, Germany), with a sample rate of 120 Hz and accuracy of over 1 degree of visual angle. The eye-tracker recorded the data from both eyes from the reflection of near-infrared light off the cornea and pupil. It was positioned in front of the subject just below the 22-inch flat-screen monitor where the stimuli were presented using SMI Experiment Center Software. The distance from the screen and the inclination angle of the system were adjusted for each toddler to obtain the best possible eye-tracking acquisition. The placement suggestions provided by SMI iViewX Software were used for the correct positioning of the eye-tracker. The child was seated on a parent’s lap at an approximate distance of 50 cm from the screen. Before starting the experimental task, a five-point calibration sequence was run. A cartoon was chosen for calibration to maximize the toddlers’ attention to the screen.

### 2.4. Measures

The measurements were computed only for the JA segment of the two tasks. Overall, the looking time on screen, alternating gaze behavior, transitions and fixations on areas of interest (AOIs) were considered as measures for analysis. The same measures used in our previous study [28] were used for comparison. Moreover, we added another measure introduced by Thorup et al. [30], i.e., the alternating gaze, which computed the mean number of gaze alternations between two AOIs. The following area of interest (AOI) were selected using SMI BeGaze Software (SensoMotoric; Teltow, Germany): the model’s face, target object and non-target object. The transitions and alternating gazes between these AOIs were computed. The measurements referring to transitions and alternating gaze were computed by extracting raw data and analyzing them in Matlab (MathWorks, Natick, MA, USA) using homemade scripts.

In addition, the fixation duration, expressed as a percentage, was computed for each of this AOI using BeGaze Software by SMI. To avoid counting unconscious looking, a fixation threshold of 60 milliseconds was applied to the raw data, as already performed in the study by Falck-Ytter et al. [51] on toddlers.

The description of all the computed measure is reported in Supplementary Table S1.

### 2.5. Statistical Analyses

Statistical analyses were performed in Statistical Packages for Social Sciences (SPSS, Version 22, IBM Corp., Armonk, NY, USA).

A comparison of the ASD, HR-noASD and TD populations in terms of age, sex, cognitive level and T score at the CBCL was performed using an analysis of variance (ANOVA). A comparison between toddlers with ASD and HR-noASD was performed using t Student on the ADOS-Calibrated Severity Score (CSS), levels of adaptive functioning (VABS-II), SCQ score, number of words understood or produced and gestures (MacArthur Questionnaire).

For the eye-tracking measures, the Shapiro–Wilk test was initially applied to test the normality of the variables. Given that the three groups were different in terms of Griffiths performance, this measure was used as covariate in all the analyses. For normal variables, an ANCOVA was applied. When a non-parametric test was required, variables and covariates were transformed into ranks and the analysis of covariance was performed on ranks. Effect sizes were estimated by partial eta squared (η^2^; values between 0.01 and 0.06 are generally considered a small effect, between 0.06 and 0.14 a medium effect and those above 0.14 are regarded as a large effect). Pearson correlations or Spearman correlations, according to the distribution of the variables, were used to examine correlations among eye-tracking measures and ADOS_CSS, ADOS_SA-CSS, ADOS-RRB-CSS and the following items selected from ADOS-2 and ADOS-G: showing, pointing, gesturing, eye contact, initiating joint attention in the HR-noASD and ASD groups.

## 3. Results

### 3.1. Overall Looking Time on Screen

The three groups were not significantly different in terms of overall looking time on screen, i.e., the total time that the children spent looking at the screen during the joint attention segment (F = 0.486, *p* = 0.816, η^2^ = 0.041).

### 3.2. Initiating Joint Attention

The results in the initiating JA-1 and JA-2 tasks are shown in Table 2 and Table 3, respectively. In the initiating JA-1 task, the three groups were not significantly different in terms of fixation duration for face (F = 0.059, *p* = 0.94), target object (F = 0.176, *p* = 0.83) and non-target object (F = 0.794, *p* = 0.45). Furthermore, the analyses revealed that HR-noASD subjects performed a lower number of transitions from the target object to face as compared with the ASD population (2.37 ± 2.09, *p* = 0.002 and 3.06 ± 1.61, *p* = 0.11, respectively). The difference between HR-noASD and ASD subjects in the number of transitions from face to target object was significant (*p* = 0.016), whereas there was no significant difference between TD and HR-noASD groups (*p* = 0.11).

**Table 2:** Transitions, alternating gaze and fixation for the joint attention phase of the IJA-1.

	Groups			Groups Comparison			Post-Hoc Comparisons	
	HR-noASD (*N* = 19)	ASD (*N* = 17)	TD (*N* = 16)	F	*p* Value	η^2^	HR-noASD vs. ASD (*p* Value)	HR-noASD vs. TYP (*p* Value)
IJA1								
FD F (%)	30.52 ± 31.18	29.65 ± 23.14	22.71 ± 17.76	0.059	0.94	0.003	0.74	0.85
FD TO (%)	22.73 ± 17.47	20.64 ± 11.91	27.72 ± 24.86	0.176	0.83	0.008	0.84	0.67
FD NTO (%)	9.03 ± 10.58	5.60 ± 5.30	6.76 ± 6.65	0.794	0.45	0.047	0.23	0.39
Transitions TO → F	2.37 ± 2.09	4.82 ± 2.74	3.06 ± 1.61	6.261	0.004 *	0.218	0.002 *	0.72
Transitions F → TO	2.72 ± 2.32	4.88 ± 2.78	4.31 ± 1.40	3.270	0.048 *	0.140	0.016 *	0.11
Transitions NTO → F	0.17 ± 0.39	0.75 ± 0.77	1.12 ± 1.25	4.210	0.02 *	0.158	0.07	0.008 *
Transitions F → NTO	0.08 ± 0.28	0.37 ± 0.61	0.62 ± 0.95	1.469	0.24	0.068	0.18	0.12
Transitions TO → NTO	1.09 ± 1.70	1.64 ± 1.53	2.93 ± 1.91	3.260	0.049 *	0.140	0.10	0.015 *
Normalized Transition Score	0.80 (0.38)	0.56 ± 0.56	0.52 ± 0.45	2.289	0.11	0.096	0.17	0.04 *
Alternating gaze F ↔ TO	1.06 ± 0.97	2.52 ± 1.32	1.84 ± 0.71	8.554	0.01 *	0.800	<0.001 **	0.13
Alternating gaze F ↔ NTO	0.05 ± 0.10	0.28 ± 0.30	0.43 ± 0.52	3.878	0.028 *	0.150	0.06	0.012 *
Alternating gaze NTO ↔ TO	0.19 ± 0.37	0.41 ± 0.38	0.73 ± 0.48	4.888	0.012 *	0.178	0.10	0.003 *

Abbreviations: IJA-1, initiating joint attention 1; HR-noASD, high risk-no Autism Spectrum Disorder; ASD, Autism Spectrum Disorder; TD, typical development; SD, standard deviation; FD, fixation duration; TO, target object; NTO, non-target object. * *p* < 0.05, ** *p* < 0.001.

**Table 3:** Transitions, alternating gaze and fixation for the joint attention phase of the IJA2.

	Groups			Groups Comparison			Post-Hoc Comparisons	
	HR-noASD (*N* = 19)	ASD (*N* = 17)	TD (*N* = 16)	F	*p* Value	η^2^	HR-noASD vs. ASD (*p* Value)	HR-noASD vs. TYP (*p* Value)
IJA2								
FD F (%)	21.77 ± 18.62	24.28 ± 18.52	15.02 ± 11.25	0.202	0.82	0.009	0.76	0.69
FD TO (%)	22.60 ± 16.03	32.53 ± 17.61	36.07 ± 18.09	1.513	0.23	0.074	0.12	0.21
Transitions TO → F	2.82 ± 2.18	6.11 ± 2.17	3.71 ± 2.19	9.837	<0.001 **	0.309	<0.001 **	0.34
Transitions F → TO	3.83 ± 1.89	5.41 ± 2.37	3.71 ± 1.77	1.944	0.15	0.091	0.08	0.91
Alternating gaze F ↔ TO	1.38 ± 1.12	2.88 ± 1.09	1.62 ± 1.08	8.57	0.001 *	0.272	<0.001 **	0.62

Abbreviations: IJA-2, initiating joint attention; HR-noASD, high risk-no Autism Spectrum Disorder; ASD, Autism Spectrum Disorder; TD, typical development; SD, standard deviation; FD, fixation duration; TO, target object; NTO, non-target object. * *p* < 0.05, ** *p* < 0.001. Moreover, post hoc analyses showed that alternating the gaze between the target object and the model’s face was statistically different between HR-noASD and ASD subjects (*p* < 0.001), with higher values for the ASD population (Figure 1). Alternating the gaze between the non-target object and the model’s face, and between the non-target and target objects differed significantly between the HR-noASD and TD groups (*p* = 0.012 and *p* = 0.003, respectively), with lower values for the HR-noASD population (Figure 2).

A significant trend in the same direction was detected for the number of transitions from the non-target object to face (*p* = 0.008) as well as from the target object to the non-target object (*p* = 0.015) when HR-noASD subjects were compared with the TD population. The normalized transition score was significantly higher in the HR-noASD group as compared with TD subjects (*p* = 0.04).

**Figure 1:**
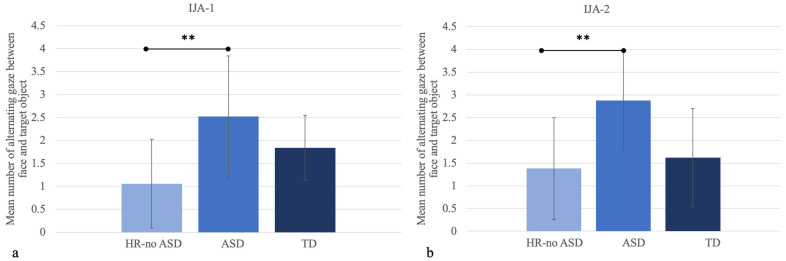
Number of gaze alternations made between the model’s face and the target object in IJA-1 (**a**) and IJA-2 (**b**). Error bars represent standard deviations. ** *p* < 0.001.

In the initiating JA-2 task with an unpredictable event, no difference between the groups was found for fixation duration on the face (F = 0.202, *p* = 0.82) and target object (F = 1.513, *p* = 0.23). Post hoc analyses revealed that the number of transitions from the target object to face and gaze alternations between the face and target object differed significantly between HR-noASD and ASD subjects (*p* < 0.001 and <0.001, respectively) (Figure 1). No group difference was found in the number of transitions from face to the target object (F = 1.944, *p* = 0.15).

According to the ANCOVA, this was not the main effect of performance quotient on alternating gaze between target object and face or on alternating gaze between not-target object and face during IJA-1 and IJA-2 tasks.

### 3.3. Correlations between Eye-Tracking and Clinical Measures in HR-noASD and ASD

**Figure 3:**
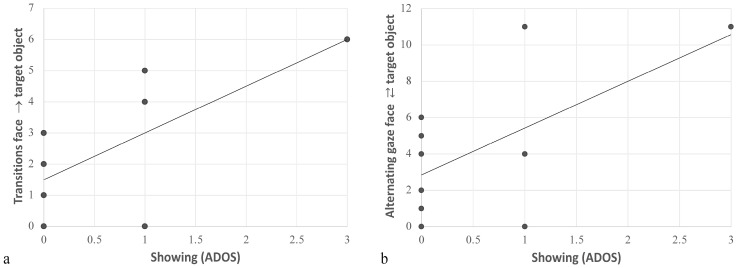
Correlation between Showing and (**a**) transitions from face to target object and (**b**) alternating gaze between face and target object in HR-noASD population (IJA-1).

In the initiating JA-1 task for the HR-noASD group, the ADOS_Showing item was positively correlated with the number of transitions from face to both target object (r = 0.69, *p* = 0.025) and negatively correlated with transition from face to non-target object (r = −0.74, *p* = 0.013). This finding indicated that frequent transitioning from face to non-target objects was associated with greater impairment in Showing, a clinical measure of IJA (Figure 3).

In the ASD population, a negative correlation was found between the number of transitions from the target object to face and ADOS_Initiating JA (r = −0.57, *p* = 0.018). The latter result indicates that the fewer the transitions from the target object to the face are, the greater the struggle to initiate JA in real life.

In IJA task 2, no significant correlations were found between eye-tracking and clinical measures in the HR-noASD. Conversely, both the total Calibrated Severity Score (CSS) and Social Affect—Calibrated Severity Score (SA–CSS) were negatively correlated with transitions from the target object to face (r = −0.69, *p* = 0.002 and r = −0.063, *p* = 0.006, respectively) as well as with alternating gaze between target object and face (r = −0.66, *p* = 0.004 and r = −0.58, *p* = 0.015, respectively) for the ASD population. This finding indicated that reduced gaze alternations between target object and face were associated with elevated symptoms of ASD, in particular with SA impairment.

## 4. Discussion

As far as phenotypic characteristics, our results indicated that the non-verbal cognitive skills of HR-noASD subjects turned out to be poorer than those of low-risk toddlers and similar to those of ASD children, with mean scores at the lower limits of the normal range. This finding is in line with some previous studies [10,52,53]. Indeed, Charman and colleagues [53] have demonstrated that the occurrence of developmental delay at 36 months is three times higher in high-risk siblings without ASD than in LR children. Of note, in contrast to the abovementioned studies, which compared the global intellectual quotient, we focused only on non-verbal cognitive skills.

Secondly, the HR-noASD toddlers showed better global adaptive functioning as compared with ASD subjects, in particular in socialization and communication skills. Similar findings have emerged from a recent investigation that compared VABS scores between concordant and discordant siblings and detected better adaptive skills in all domains in HR-noASD than in siblings with ASD [54]. Conversely, a recent prospective study by Salomone et al. [10] reported that at 36 months, VABS adaptive behavior composite (ABC) scores were lower in both HR-ASD and HR-noASD children than in LR children and that the two high-risk groups did not differ from each other in the adaptive abilities. However, a comparison between our investigation and the two aforementioned studies requires some caution since we focused on toddlers referred to clinical centers for ASD symptoms who received an early ASD diagnosis (mean age: 24.6 months), whereas the other two assessed high-risk siblings with ASD, which have been clinically monitored starting from the first months of life by a research team.

Taken together, these findings suggest that early low-level adaptive functioning and reduced non-verbal cognitive skills may be related to BAP in HR-noASD siblings as compared with LR controls, as already reported at 36 months by Charman et al. [53] and Messinger et al. [52]. Hence, primary care pediatricians and child psychiatrists have an important role in conducting developmental surveillance for all younger siblings of children with ASD until mid-childhood and beyond [16,55].

The evaluation of symptom severity by the ADOS calibrated severity score (CSS) [39] revealed that the HR-noASD group showed better social and communicative skills than ASD subjects but, at the same time, that these toddlers displayed high levels of restricted and repetitive behaviors (RRB). This latter finding is in line with previous studies that failed to find significant differences in the rate of RRB in siblings of ASD children with and without a subsequent ASD diagnosis [56,57]. In more detail, HR-ASD and HR-noASD toddlers did not significantly differ in the repetitive use of objects [58,59] and in the repetitive body movement [58].

Longitudinal studies in large samples have also revealed that at 36 months, HR-noASD children exhibited higher levels of RRB, though without social affect difficulties, as compared to the low-risk group. Furthermore, boys exhibited higher levels of RRB than girls [52], since boys predominated in our HR-noASD sample, this issue may partially account for the high mean RRB-CSS score we detected.

Regarding the linguistic profile, HR-noASD children had better vocabulary comprehension and production and a broader range of gestures as compared with the ASD group. The literature on the development of gestures in HR-noASD and HR-ASD subjects is inconclusive. Some studies have detected lower gestures in HR-ASD in their second year and as early as 12 months [60,61], while Goldberg et al. [62] found no differences between the two groups. Moreover, the HR-noASD group had a wider receptive and productive vocabulary than the ASD children. Previous investigations on this topic revealed that HR-noASD toddlers are at an increased risk of language delays, with a more pronounced impairment in receptive than expressive language [63]. More broadly, the findings reported in the literature on language development are also inconsistent, with some studies that highlighted an increased rate of language delay in HR [12,64], while others revealed no significant differences between HR and LR toddlers [53]. A recent meta-analytic review observed worse receptive and expressive language skills in siblings of children with ASD compared to siblings of children with TD [65]. Chita-Tegmark et al. [66], using an eye-gaze measure of receptive language, detected that children at heightened risk for ASD had a significantly lower accuracy at 36 months, but not at 18 or 24 months, as compared with the low-risk control group. Moreover, no significant group difference was found in terms of speech processing. These findings suggest that high-risk ASD children might have difficulties forming more robust lexical representations of words using communication and social skills. One possible explanation for these contradictory results is that HR infants without ASD do not represent a homogeneous group [7] since some are indistinguishable from LR peers, some display early but transient delays in communication development and others exhibit a clinically significant delay in receptive or expressive language [67]. At present, the question of whether language delay should be considered a part of the BAP remains open.

As far as the eye-tracking experiment, the results of this study indicated that high-risk-noASD, ASD and TD toddlers did not display significant differences in terms of looking time (fixation duration) on the face, on the target object and not-target object during the initiating joint attention (IJA) task. Conversely, other investigations performed at an earlier age highlighted different findings. Specifically, attenuated reduction in looks to faces between 9 and 15 months of age in the HR group compared with the LR group [68], reduced overall looking time at images of faces in six months old high-risk–noASD than both high-risk-ASD and controls [17], and less time looking at the adult’s face immediately after the onset of direct gaze initiation in 10-month-olds HR group as compared to TD group were reported [69].

On the other hand, in the current study, some significant differences were found among the three groups in terms of alternating gaze, a measure of looking performance recently reported by Thorup et al. [30]. In particular, IJA-1 (the initiating joint attention task with a predictable event) proved better able than IJA-2 (with an unexpected event) to reveal the differences among the three groups as well as to characterize the visual pattern of each group. This finding could be partly ascribed to the presence in this task of the non-target object in addition to the target object.

More in detail, in IJA-1, the HR-noASD children behaved similarly to TD toddlers and performed fewer gaze alternations between the model’s face and the target object with respect to the ASD group. Regarding alternating gaze between the model’s face and the non-target objects, HR-noASD and ASD toddlers had similar results: i.e., they alternated less frequently than the TD group. Correlation analyses revealed that lower transitions from a target object to a face in the ASD group were associated with a greater impairment in initiating JA in real-life situations (IJA_ADOS).

The results of the IJA-2, in which there is an absence of a non-target object, confirmed that HR-noASD and TD subjects exhibited the same looking performance with regard to the target object. Indeed, both groups alternated their gaze between the model’s face and target object less often than the ASD group. Moreover, this reduced frequency of gaze alternations between face and target object was associated with more core ASD symptoms (total CSS). Specifically, when we split the social affect (SA) and the repetitive and restricted behavior (RRB) domains, only SA showed a negative correlation with gaze alternations between the face and target object, indicating that increased alternations between face and target object are associated with less impairment of social and communicative abilities, but not of restricted and repetitive behaviors. The fact that the HR-noASD subjects showed far better social and communicative behaviors than the ASD children but high levels of RRB might explain why the HR-noASD behave similarly to the TD group, but differently from the ASD subjects, in terms of gaze alternations between face and target object.

Regarding the alternating gaze from the face to the non-target object and between the still object and moving object, HR-noASD showed a pattern similar to that of the ASD group. Although we did not find a significant relation between face-to-non-target-object alternating gaze and RRB-Calibrated Severity Score (ADOS), we may hypothesize that an atypical visual process is involved in this phenomenon. Indeed, there is extensive literature on atypical multisensory processing in ASD subjects [70,71], which has explored whether abnormal sensory domains, in addition to being a core phenotypic marker of autism, belong to the BAP [72]. In fact, alterations in sensory processing are more frequent in the parents [73] and siblings [74] of ASD children than in the general population. In particular, regarding visual detection, individuals with autism tend to focus on details of the perceptual world at the expense of the global percept they compose [75], and they may be less sensitive to the context [76]. Pellicano and Burr [77] have suggested a Bayesian explanation on the basis of the unusual perceptual processing in ASD. According to this hypothesis, people with ASD have weak priors (i.e., information based on previous experience); since their perception is less mediated by prior experiences, the world becomes “too real” for them. We speculated that the HR-noASD and ASD groups exhibited similar-looking performance with regard to the not-target object because they both process the world through a detail-focused perceptual style, demonstrating a relative insensitivity to a “distractor” in a scene and a relative inability to perform a general scanning of the context. Therefore, this finding could extend to the second year of life, the absence of differences in visual processing abilities between the HR-ASD and HR-noASD groups was already observed at 6 months of age [8]. An alternative explanation is that HR-noASD and ASD subjects look less often at non-target objects because they struggle to perform action prediction [78,79,80,81]. Thus, the TD children paid greater attention to the unmoving car toy because they had foreseen its possible movement.

Our results about alternating gaze between the face and target object were in contrast with a recent study by Thorup et al. [30], but the different experimental design (live interactions vs. recorded stimuli) could partially account for these discrepant findings. It is quite possible that some of the children’s looking behaviors were affected by the awareness that a person on a screen is different from a real person and that he or she was not affected by their behavior.

Overall, results from the current study confirmed our hypothesis that HR-noASD toddlers displayed a pattern of alternating gaze and transitions intermediate between that of children with ASD and children with TD. Specifically, we observed that HR-noASD subjects were more similar to ASD individuals in terms of alternating gaze (transitions) from the face to the target object, while they behaved more similarly to TD in terms of alternating gaze (transitions) from the face to non-target object. Thus, the eye tracking methodology has proven to be able to capture a possible biomarker of an early broader autism phenotype in HR-noASD individuals.

More broadly, due to its non-invasive and objective nature and its suitability for research with very young populations, the eye tracking methodology shows its potential not only in the field of early diagnosis but also in the evaluation of the intervention outcomes [82,83].

### Limitations

We acknowledge that the study has several limitations. First, the sample was too small to attempt to establish whether some significant eye-tracking variables (i.e., alternating gaze) can be used to distinguish HR-noASD from ASD and TD group subjects. Second, the control group clearly exhibited better non-verbal cognitive skills than the ASD and HR-noASD groups. An attempt to reduce the impact of this difference was made by adopting the non-verbal developmental quotient as a covariate in all comparisons. Third, the video-based eye-tracking scenario was very different from the real-life situations, and thus results should be interpreted with caution when studying social attention in response to pre-recorded stimuli and not through live interactions. Fourth, participants did not undergo an examination by an ophthalmologist to confirm the physiological visual acuity and exclude visual pathologies. However, this limitation is mitigated by the fact that all toddlers recruited in this study were followed by the primary care pediatrician who had not reported the need for an ophthalmological examination.

Future investigations should involve larger samples of subjects, not only to support results with better statistical power but also to further subgroup the heterogeneity of HR-noASD subjects on the basis of developmental trajectories. In this context, the IJA eye-tracking profile has the potential to provide an early and objective measure correlated with the clinical outcome. Such information would have several implications in clinical practice, as early detection of atypical development is a prerequisite for early intervention that, in turn, could improve outcomes and quality of life for children and their families.

## Figures and Tables

**Figure 2:**
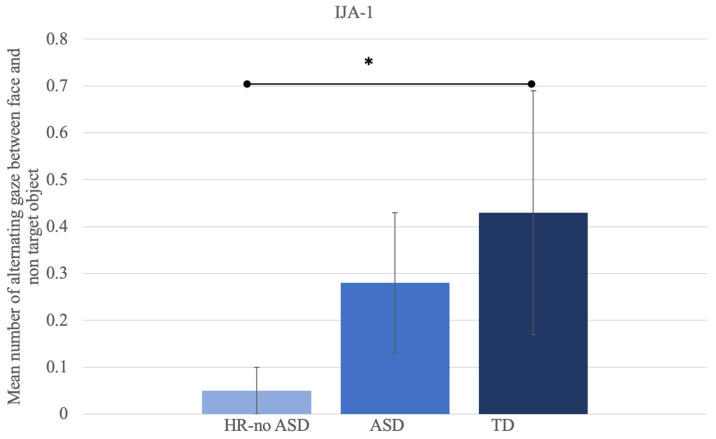
Number of gaze alternations made between the model’s face and non-target object in IJA-1. Error bars represent standard deviations. * *p* < 0.05.

**Table 1:** Demographic and clinical characteristics of ASD, HR-noASD and TD groups.

Groups				Group Comparison		Post-Hoc Comparisons		
	HR-noASD (N = 19)	ASD (*N* = 17)	TD (*N* = 16)	Comparison Coefficient	*p* Value	HR-noASD vs. ASD (*p* Value)	HR-noASD vs. TD (*p* Value)	ASD vs. TD (*p* Value)
Gender								
Boy:Girl	12:7	13:4	10:6	Χ^2^ = 0.965	0.617	-	-	-
Age (Months)								
Mean Age ± SD	22.63 ± 4.87	24.65 ± 4.24	24.88 ± 4.56	F = 1.312	0.279	0.580	0.465	1.000
Age Range	18–33	18–30	18–30	-	-	-	-	-
PQ (GMDS-ER) N = 17 N = 17 N = 16								
Mean PQ ± SD	88.59 ± 25.71	84.18 ± 22.08	110.75 ± 17.43	F = 6.384	0.004	1.000	0.023 *	0.005 *
Range PQ	35–119	35–130	83–162	-	-	-	-	-
SCQ (last 3 months)	*N* = 13	*N* = 17	-					
Raw Score ± SD (range)	5.08 ± 3.84 (2–16)	12.18 ± 4.42 (3–20)	-	t = 4.6	0.0002 **	-	-	-
ADOS	*N* = 14	*N* = 17						
Total CSS ± SD (range)	1.71 ± 0.91 (1–4)	6.59 ± 1.42 (4–9)	-	t = 11.1	0.0001 **	-	-	-
SA-CSS ± SD (range)	1.43 ± 0.65 (1–3)	6.71 ± 1.53 (3–10)	-	t = 12.02	0.0001 **	-	-	-
RRB-CSS ± SD (range)	4.57 ± 2.79 (1–9)	6.47 ± 2.87 (1–10)	-	t = 1.85	0.73	-	-	-
VABS-II	*N* = 12	*N* = 14						
ABC Score ± SD (range)	95.50 ± 15.39 (58–115)	79.54 ± 6.51 (70–95)	-	t = 3.42	0.02 *	-	-	-
Communication Score ± SD (range)	93.75 ± 11.33 (71–115)	70.36 ± 16.48 (21–90)	-	t = 4.14	0.0003 **	-	-	-
Daily Living Skills Score ± SD (range)	96.42 ± 21.07 (43–121)	82.50 ± 16.5 (35–103)	-	t = 1.65	0.11	-	-	-
Socialization Score ± SD (range)	96.08 ± 18.74 (44–113)	74.07 ± 15.69 (44–94)	-	t = 3.26	0.003 *	-	-	-
Motor Score ± SD (range)	98.00 ± 11.42 (85–110)	90.71 ± 15.12 (72–109)	-	t = 1.36	0.18	-	-	-
MacArthur-CDI	*N* = 13	*N* = 17						
Words Understood Raw Score ± SD (range)	231.62 ± 94.6 (0–349)	106.82 ± 92.16 (55–270)	-	t = 3.62	0.001	-	-	-
Words Produced Raw Score ± SD (range)	78.38 ± 89.80 (1–290)	11.47 ± 19.65 (0–80)	-	t = 2.99	*p* = 0.005	-	-	-
Gestures Raw Score ± SD (range)	52.23 ± 22.12 (22–115)	26.06 ± 13.42 (7–47)	-	t = 4.01	*p* = 0.0002	-	-	-

Abbreviations: HR-noASD, high risk-no Autism Spectrum Disorder; ASD, Autism Spectrum Disorder; TD, typical development; SD, standard deviation; PQ, performance quotient; GMDS-ER, Griffiths Mental Development Scale-Extended Revised; SD, standard deviation; DSM, Diagnostic and Statistical Manual of Mental Disorders; SCQ, Social Communication Questionnaire; ADOS, Autism Diagnostic Observation Schedule; CSS, calibrated severity score; SA-CSS, social affect-calibrated severity score, RRB-CSS, Repetitive and Restricted Behaviors-Calibrated Severity Score; VABS-II, Vineland Adaptive Behavior Scale Second Edition; ABC Score, Adaptive Behavior Composite Score; CDI, Communicative Development Inventory. * *p* < 0.05, ** *p* < 0.001.

## Data Availability

The data presented in this study are available on request from the corresponding author. The data are not publicly available due to ethical restrictions.

## Supplementary Materials

The following supporting information can be downloaded at: https://www.mdpi.com/article/10.3390/jpm12111789/s1, Table S1: Definitions of the joint attention measures for the two tasks (Initiating JA-1 and Initiating JA-2).
