# Supplementary material for: High-Risk Siblings without Autism: Insights from a Clinical and Eye-Tracking Study
Source: J Pers Med. 2022 Oct 29;12(11):1789. doi: 10.3390/jpm12111789 (PMC9699372; doi:10.3390/jpm12111789)
Supplement: Supplementary file 1 [file jpm-12-01789-s001.zip › jpm-1967208-supplementary.pdf]

Table S1. Definitions of the joint attention measures for the two tasks (Initiating JA-1 and Initiating JA-2).

**Measure**	**Initiating JA-1**	**Initiating JA-2**
Overall looking time on the screen (ms)	The total time that the child spent looking at the screen during the JA segment	
Fixation duration (%)	Fixation time on that Area of interest (AOI) divided by total fixation time on the screen in JA segment $\frac{FD_{AOI}}{FD_{Trial}}$	
Transitions		
face to target object (F→TO)	Subject's gaze shift from face to target object	
face to non-target object (F→NTO)	Subject's gaze shift from face to non-target object	–
target object to face (T→OF)	Subject's gaze shift from target object to face	
non-target object to face (NTO→F)	Subject's gaze shift from target object to face	–
Normalized transition score	Differences between transitions from target object to face and from non-target object to face divided by the total number of transitions from either object to face $\frac{TOF - NTOF}{TOF + NTOF}$	–
Alternating gaze		
face to target object and vieversa (F↔TO)	Sum of gaze alternations from target object to face and from face to target object	
face to non-target object and vieversa (F↔NTO)	Sum of gaze alternations from non-target object to face and from face to non-target object	
target object to non-target object and vieversa (F↔TO)	Sum of gaze alternations from target object to non-target object and from non-target object to target object	
